# Atypical cortical connectivity and visuospatial cognitive impairments are related in children with chromosome 22q11.2 deletion syndrome

**DOI:** 10.1186/1744-9081-4-25

**Published:** 2008-06-17

**Authors:** Tony J Simon, Zhongle Wu, Brian Avants, Hui Zhang, James C Gee, Glenn T Stebbins

**Affiliations:** 1 M.I.N.D. Institute, University of California, Davis, 2825 50th Street, Sacramento, CA 95817, USA; 2 Department of Radiology, University of Pennsylvania, 3600 Market St. Philadelphia, PA 19104, USA; 3 Rush University Medical Center, Department of Neurological Sciences, 1725 W. Harrison, Chicago, IL 60612, USA

## Abstract

**Background:**

Chromosome 22q11.2 deletion syndrome is one of the most common genetic causes of cognitive impairment and developmental disability yet little is known about the neural bases of those challenges. Here we expand upon our previous neurocognitive studies by specifically investigating the hypothesis that changes in neural connectivity relate to cognitive impairment in children with the disorder.

**Methods:**

Whole brain analyses of multiple measures computed from diffusion tensor image data acquired from the brains of children with the disorder and typically developing controls. We also correlated diffusion tensor data with performance on a visuospatial cognitive task that taps spatial attention.

**Results:**

Analyses revealed four common clusters, in the parietal and frontal lobes, that showed complementary patterns of connectivity in children with the deletion and typical controls. We interpreted these results as indicating differences in connective complexity to adjoining cortical regions that are critical to the cognitive functions in which affected children show impairments. Strong, and similarly opposing patterns of correlations between diffusion values in those clusters and spatial attention performance measures considerably strengthened that interpretation.

**Conclusion:**

Our results suggest that atypical development of connective patterns in the brains of children with chromosome 22q11.2 deletion syndrome indicate a neuropathology that is related to the visuospatial cognitive impairments that are commonly found in affected individuals.

## Background

Chromosome 22q11.2 deletion syndrome (22q11.2DS) is caused by a microdeletion within chromosome 22 at band q11.2 [1-3] and encompasses DiGeorge [4], Velocardiofacial [5] and several other syndromes. The phenotypic spectrum of 22q11.2DS includes over 180 physical anomalies, learning disabilities, and psychiatric manifestations [6], only a small subset of which are observed in any affected individual. Children with 22q11.2DS have significant overall reductions in brain volume of 8.5 to 11% compared to typically developing (TD) children, with white matter (WM) reductions marginally greater than those in gray matter (GM). These areas of reduced volume are concentrated in the posterior and inferior regions of the brain, including the parietal, temporal and occipital lobes, and in the cerebellum. When total brain volume is accounted for, the frontal lobes are relatively enlarged [7,8].

Our recent study [9] of 18 children with 22q11.2DS and 18 TD controls replicated the reductions in total (8.9%), WM (11.07%) and GM (9.9%) volumes in the 22q11.2DS group as well as key regional GM reductions in occipital, parietal, temporal and cerebellar areas and detected two regions of increased frontotemporal GM volume. Cerebrospinal fluid (CSF) analyses detected clusters of increased volume in the 22q11.2DS group that co-localized with many medial GM reductions and indicated lateral and fourth ventricle dilation. Diffusion tensor imaging (DTI) results showed that TD controls had higher fractional anisotropy (FA) values, usually taken to indicate greater integrity and/or orientation of major fiber tracts, in an area encompassing the corpus callosum, some cingulate white matter, and the posterior thalamic pulvinar region. The 22q11.2DS group had higher FA values in a cluster encompassing anterior to posterior cingulate gyrus and extending into the splenium of the corpus callosum, the precuneus and portions of the inferior parietal lobe. One cluster of increased FA, in the right supramarginal gyrus region, was also detected. The finding was consistent with that of Barnea-Goraly et al. [10].

We interpreted these different FA patterns as indicating that lateral ventricle dilation in the 22q11.2DS group is related to changes in the location and morphology of the corpus callosum. These may have negatively impacted parietal, or even wider, connectivity. We recently reported [11] that regional callosal area related differently to performance in children with 22q11.2DS and TD controls on an attentionally demanding counting task. This suggests that affected children may be using a different, atypical, cortical network in this task, which might partially explain their difficulties. Our current study explores the functional significance of connectivity changes for impairments in visuospatial attention [12,13] in DS22q11 by calculating from our previous diffusion tensor data [9] several diffusivity measures and correlating them with cognitive performance measures. While the resolution of our original data set is rather limited and does not lend itself to the newest analytical approaches or afford good visualization of results, we have introduced cutting edge registration and normalization methods that we believe increase the accuracy of the analyses considerably. Thus, we felt strongly that we should use the same data to first explore the relationship between diffusion scalars and behavior. This is because it would allow us to carry out the most direct test of our hypothesis about the functional implications of connectivity changes by holding the scanner characteristics and participant characteristics constant. Having relocated the research project to a new institution (University of California, Davis), we have been acquiring higher resolution data on a 3 Tesla scanner with different participants and this will be subsequently analyzed to determine if the results presented here are replicated.

## Methods

### MRI protocols and participants

Participants, aged 7–14 years, were 18 children with 22q11.2DS (mean age 9.8, S.D. = 1.4, 11 female), and 18 TD children (mean age 10.4, S.D. = 1.9, 7 female). Children with 22q11.2DS were recruited through the "22q and You" center at The Children's Hospital of Philadelphia. Diagnosis of 22q11.2DS was defined as a positive result from the standard fluorescence in situ hybridization (FISH) test. For all participants, parental consent and child assent was given in accord with the requirements of the Institutional Review Board of the Children's Hospital of Philadelphia.

Before MRI scanning, all subjects underwent acclimation and head motion suppression training in a mock MRI scanner. All subjects were scanned on a 1.5T Siemens MAGNETOM Vision scanner. For each child, high-resolution three-dimensional structural MRI and diffusion tensor MRI data were acquired. We used a T1-weighted magnetization prepared rapid gradient echo (MP-RAGE) sequence with the following parameters: TR = 9.7 ms, TE = 4 ms, flip angle = 12°, 160 slices in sagittal plane; 256 × 256 matrix; field of view 256 × 256 mm^2^, giving a voxel size of 1.0 × 1.0 × 1.0 mm, and a single-shot, spin-echo, diffusion-weighted echo-planar imaging (EPI) sequence with the following parameters: 20 contiguous 5 mm-thick, axial slices with in-plane resolution of 2 × 2 mm, TR/TE = 6000/100 ms, matrix size = 128 × 128 and field of view 256 × 256 mm. The diffusion scheme was a single non-diffusion-weighted (*b *= 0s/mm^2^) reference image followed by six diffusion-weighted images measured with unique non-collinear diffusion encodings at gradient directions *YZ*, *XY*, *XZ*, *Y-Z*, *X-Y*, and *X-Z *having *b *= 1000s/mm^2^. Since whole brain coverage was not possible with this sequence, the slab was placed to cover the more superior portions of the brain, generally from the top of the brain to the superior third of the cerebellum. A neuroradiologist examined the images for motion artifacts and corrupted images. Three DTI scans were unusable due to excessive motion, image corruption, or small field of view, resulting in analyses of 16 children with 22q11.2DS and 17 TD controls.

### Measures of diffusion tensor imaging

The diffusion-weighted images were first corrected for eddy current distortion using the FSL Diffusion Toolkit by affine registration of all of the images to the non-diffusion weighted image. The 3 × 3 diffusion tensor was estimated for each voxel location within a brain tissue mask generated from the non diffusion weighted image using the FSL Brain Extraction Tool [14]. For each diffusion tensor, the associated eigenvalues (*λ*_1_, *λ*_2_, *λ*_3_) and eigenvectors (*e*_1_, *e*_2_, *e*_3_) were calculated. The former values were used to obtain voxelwise maps of the following diffusion indices [15]: fractional anisotropy (FA) ($\left(FA=\sqrt{\frac{3}{2}}\frac{\sqrt{{\left(\lambda_{1}-\lambda \right)}^{2}+{\left(\lambda_{2}-\lambda \right)}^{2}{\left(\lambda_{3}-\lambda \right)}^{2}}}{\sqrt{\lambda_{1}^{2}+\lambda_{2}^{2}+\lambda_{3}^{2}}}\right)$), mean diffusivity (MD) (MD = (*λ*_1 _+ *λ*_2 _+ *λ*_3_)/3), axial diffusivity (AD) (*AD *= *λ*_1_), and the radial diffusivity (RD) (*RD *= (*λ*_2 _+ *λ*_3_)/2). MD represents a measure of the translational mobility of intracellular and extracellular fluid diffusion. AD represents a measure of the fluid diffusivity in the orientation parallel to the principle direction of the tensor. RD represents a measure of the fluid diffusivity in the plane perpendicular to the principle direction of the tensor. Finally, FA represents a measure of intravoxel coherence of water diffusion, and in white matter this marker is typically used as a measure of tract integrity. Group comparisons of the DTI-derived maps were performed by spatially normalizing each map to a common anatomic template.

### Image normalization and smoothing

Atlas-based normalization enables a cross-sectional comparison of diffusion and tissue related measures in our TD and 22q11.2DS populations, as well as correlation with cognitive measures. Our normalization procedure first rigidly aligns individual b0 images to individual T1 images using the mutual information similarity metric. This step brings both T1 and diffusion-related neuroanatomical images into a common individual space. Each individual image is then mapped to an optimal, dataset-specific shape and appearance template space, derived by a diffeomorphic groupwise normalization method [16,17]. Optimal local templates are known to improve normalization accuracy and precision, particularly in patient data [18]. The final stage of our processing involves transferring the normalized data to the standard Montreal Neurological Insititute (MNI) template space for prior-based tissue segmentation, Talairach coordinate localization and statistical analysis.

**Figure 1:**
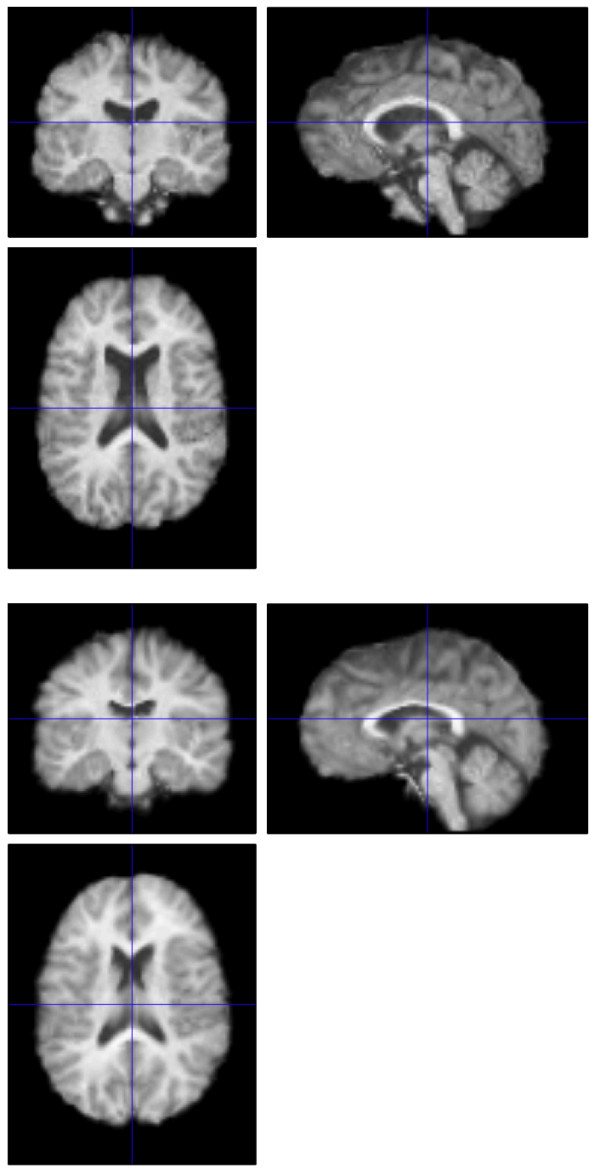
Comparison of normalization of a single child with 22q11.2DS to the CCHMC2 template using SPM5 (top) and the high dimensional methods utilized in the present study (bottom).

**Figure 2:**
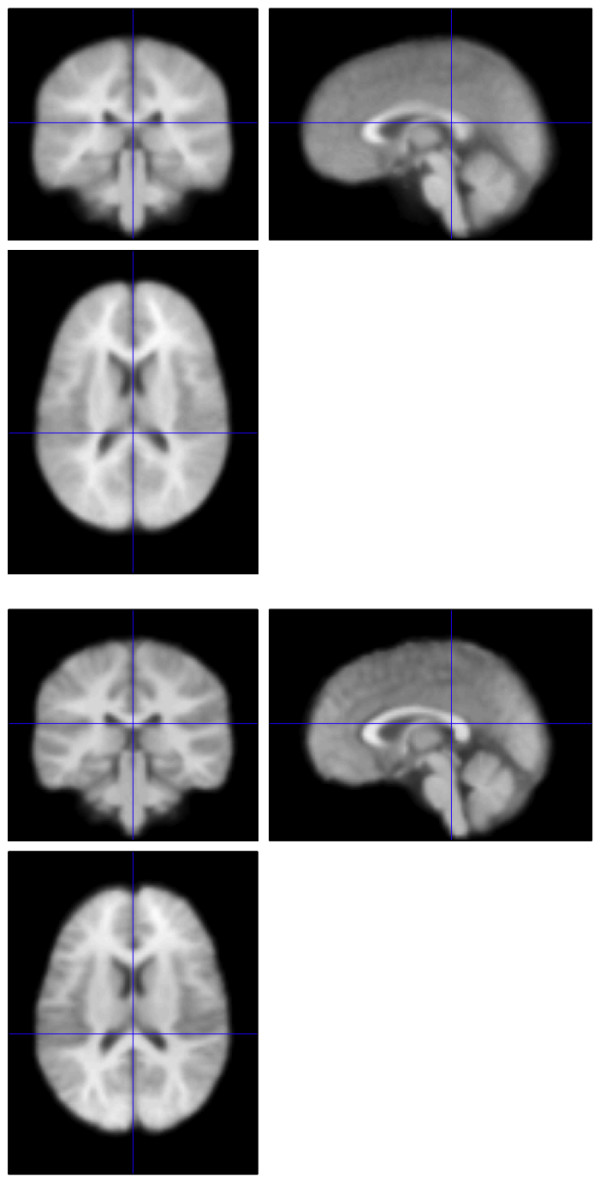
Comparison of the population template of all 36 children in the current study resulting from SPM5 (top) and the high dimensional methods utilized in the present study (bottom).

This procedure improves upon our previous analysis [9] that used the pediatric CCHMC2 template (Cincinnati Children's Hospital Medical Center, Cincinnati, OH) as the target anatomical space for group normalization via SPM99. The CCHMC2 template was constructed by normalizing the brains of 200 children aged 5 to 18 years old to the MNI template. Although these were the most advanced methods available at the time, registration accuracy suffered due to significant residual shape differences within the brains of the 22q11.2DS and TD children. The current study uses improved normalization methods together with a dataset-specific template. Specifically, the SyN (symmetric normalization) algorithm yields a high dimensional, intrinsically symmetric, diffeomorphic registration that guarantees no topological folding occurs while capturing large deformation differences between neuroanatomy. The method has performed well in comparison to state of the art methods on challenging neurodegenerative [19] and pediatric [20] datasets. SyN uses two strategies to guarantee that normalization results are not biased with respect to template choice. First, the normalization parameters for each individual registration are not affected by which image is selected as the "fixed" or reference template space [19]. Second, the method estimates, during a group-wise iterative optimization process, the most representative neuroanatomy in terms of shape and appearance [16,21]. In this way, we obtained a T1 MRI template representative of the average neuroanatomy of all 36 participants in our study that is closer in terms of shape, size and internal configuration than any other existing template. Moreover, due to high quality normalization, the template also retains high contrast and sharp features in comparison to standard templates. Figure 1 illustrates the difference between an SPM5 normalization of a single brain from a child with 22q11.2DS to the CCHMC2 template (top) and the normalization resulting from the methods described above. Figure 2 presents a comparison of the population template resulting from an SPM5 normalization (top) and that resulting from the methods described above (bottom). As these figures demonstrate, we believe that the use of high-dimensional warping allows us to optimally map the same anatomy from very different brains (here from children with 22q11.2DS and their typically developing counterparts) together. Therefore, when we report differences in diffusion measures, we have much greater confidence that these differences truly reflect changes in the same regions across participants than was true in our previous analysis [9].

To enable us to report our results in standard space we carried out the following mappings. After normalizing to the population template, the registration algorithms of SPM5 were used to map our results to the pediatric CCHMC2 template. The individual maps of DTI measures were also transferred to the standard space using the transformations from the population template to the CCHCM2 template. The resulting images have the resolution of 2 × 2 × 2 mm. A common white matter mask for the TD and 22q11.2 populations was computed from tissue densities of gray matter (GM), white matter (WM) and cerebrospinal fluid (CSF), which were segmented from individual normalized T1 images using SPM5. All GM/WM/CSF images were averaged and the common white matter mask was calculated as (*i*2 > *i*l) & (*i*2 > *i*3) & (*i*2 > (1- *i*l - *i*2 - *i*3)) where i1, i2, and i3 represent the average probability maps of GM, WM and CSF. Because the DTI coverage was not sufficient to acquire data from the whole brain, i.e. the normalized maps were a subset of the entire brain area that was missing the most inferior regions, a mask of DTI common coverage for all participants was also created. The two masks were combined and used in the majority of analyses except for the diffusivity analyses, which used only the DTI mask.

Spatial smoothing is required in order to improve signal-to-noise ratio and the sensitivity of statistical analysis since the image processing steps are expected to introduce noise in the measures [22]. It is also necessary because multiple test voxel-based analyses assume that the underlying data distributions approximate continuous Gaussian random fields [23]. Previously we used a 12 mm FWHM isotropic Gaussian kernel [9] with the CCHMC2 template and SPM99 normalization. Here we used a 6 mm FWHM smoothing kernel to exploit the high-resolution detail afforded by the combination of our template and the high quality normalizations obtained. The choice was also based on the expectation of finding relatively focal effects, and thus a smaller smoothing kernel would be more likely to detect these with a greater signal strength.

## Results

### Voxel-Based statistical analysis

Voxel-based statistical analysis was carried out by comparing the voxelwise data values of the two groups using two-sample *t *tests based on the general linear model [24]. While all analytical methods face some limitations, we chose to use this method for consistency with our previous analyses. Also, our use of advanced registration methods and a population template to minimize normalization difficulties increased our confidence in the accuracy of those registrations and thus the validity of the results we obtained. For simplicity, we use the abbreviations TD and DS (22q11.2DS) in this section. Using SPM5 we analyzed, for each normalized map, both the "TD minus DS" and "DS minus TD" contrasts. Anatomical locations were translated to Talairach coordinates via established linear transformations. Significance levels for the t statistic were controlled by the false discovery rate (FDR) for multiple comparisons and we report clusters that exceeded the conservative extent threshold of 50 voxels and uncorrected voxel level significance at p < 0.001 with a further criterion of exceeding the FDR statistic at the corrected voxel level threshold of p < .05.

**Figure 3:**
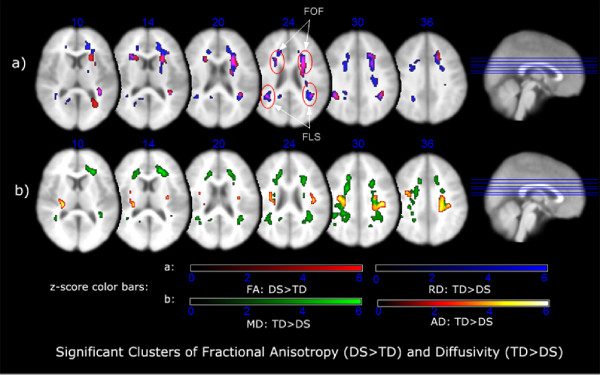
Axial slices of clusters overlaid on T1 template for FA of DS > TD, and diffusivities of TD > DS. The common areas are arrowed for the FA of DS > TD and RD of TD > DS.

Analyses detected significant clusters for all diffusivity measures with the exception of the FA (TD > DS) contrast. Table 1 presents the coordinates of the peak Talairach locations of clusters where children with 22q11.2DS had higher fractional anisotropy values than typical children while Table 2 presents the coordinates of the peak Talairach locations of clusters where typical children had higher radial diffusion values than children with 22q11.2DS. Figure 3a shows overlays of all significant clusters for the FA(DS > TD) and RD(TD > DS) contrasts and 3b shows the MD(TD > DS) and AD(TD > DS) contrasts. The most striking result is seen in Fig. 3a slice z = 24 where the FA(DS > TD) and RD(TD > DS) clusters are almost completely co-localized (creating pale purple common clusters). Neighboring slices show the inferior and superior extent of the overlap. This pattern suggests that the two groups had complementary patterns of neural connectivity in these specific bilateral parietal and frontal locations. Clearly, to confirm the differences in connective patterns in these clusters it would be desirable to visualize the tensors using fiber tracking or some other method. Unfortunately, the resolution of our data was not sufficient to offer any clear visualization and this is an analysis that will only be possible with our newer data set.

**Table 1:** Main clusters and *subclusters *in DS > TD of fractional anisotropy

Brain area	Cluster size	Z score	MNI coordinates
	(2 mm^3 ^voxels)	(voxel level)	(x, y, z) mm
Nucleus caudatus (NC)	551	5.42	22, 18, 10
*Fasciculus occipito-frontalis (FOF)*		4.38	22, -8, 24
Gyrus temporalis superior (GTs)	87	4.8	-38, -48, 20
Fasciculus longitudinalis superior (FLS)	185	4.69	38, -40, 12
*Fasciculus longitudinalis superior (FLS)*		4.26	40, -44, 28
Nucleus caudatus (NC)		3.71	32, -30, 4
Fasciculus occipito-frontalis (FOF)	131	4.44	-22, 18, 18
*Fasciculus longitudinalis superior (FLS)*		3.53	-30, 22, 14
*Fasciculus longitudinalis superior (FLS)*		3.45	-30, 12, 18

**Table 2:** Main clusters and subclusters in TD > DS of radial diffusivity

Brain area	Cluster size	Z score	MNI coordinates
	(2 mm^3 ^voxels)	(voxel level)	(x, y, z) mm
Sulcus callosomarginalis (Scm)	1387	5.58	18, 38, 12
*Fasciculus longitudinalis inferior (FLI)*		5.57	38, -42, 10
*Fasciculus occipito-frontalis (FOF)*		5.21	24, 10, 22
Fasciculus longitudinalis superior (FLS)	227	4.81	-38, -46, 26
*Radiatio optica (Ro)*		4.45	-38, -46, 8
*Lobulus parietalis inferior (LPi)*		3.25	-52, -44, 38
Tapetum (T)	138	4.69	-24, -52, 36
*Tapetum (T)*		3.71	-20, -44, 28
*Forceps major (Fm)*		3.62	-18, -56, 10
Forceps major (Fm)	465	4.68	-18, -56, 10
*Fasciculus occipito-frontalis (FOF)*		4.44	-22, 4, 28
*Gyrus cinguli (GC)*		4.17	-20, 2, 44
*Sulcus callosomarginalis (Scm)*	73	3.69	-22, -24, 46
*Sulcus callosomarginalis (Scm)*		3.63	-26, -32, 46

### ROI-Based statistical analysis

**Figure 4:**
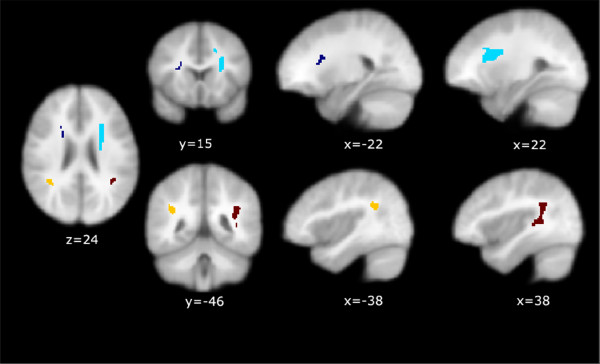
Location and extent of overlapping ROIs derived from FA (DS > TD) and RD (TD > DS) contrasts in the SPM analysis.

To further analyze this pattern, ROI based analyses were performed. The boundaries of the clusters were proscribed using the MarsBAR tool by including every suprathreshold voxel in the clusters from the FA (DS > TD) and RD (TD > DS) contrasts. The locations and extent of these ROIs are presented in Figure 4. Table 3 presents the coordinates of the peak Talairach locations of those regions. Statistical analysis of the FA and RD values within the ROIs was then repeated using statistics corrected for the total number of voxels within the clusters. Results are presented in Table 4, where the group differences showing the complementary diffusion patterns are even more dramatically illustrated. Table 5 presents results of the two other variables computed, namely MD and AD. Evidence that our choice of a 6 mm smoothing kernel was close to optimal comes from the fact that repeating the analyses with a 3 mm kernel produced almost identical clusters except that they were slightly smaller in extent and with slightly reduced values of the *t *statistic.

**Table 3:** Coordinates of common regions as ROIs

Name Of ROIs	Peak/Coordinates in FA (DS > TD)	Peak/Coordinates in RD (TD > DS)	Center Coordinates – Spheres
Left FOF	4.42/(-22, 18, 18)	4.63/(-18, 26, 22)	-22, 16, 21
Right FOF	4.87/(24, 12, 20)	5.21/(24, 10, 22)	24, 9, 23
Left FLS	4.76/(-38, -48, 28)	5.97/(-38, -50, 26)	-39, -48, 27
Right FLS	4.25/(40, -44, 28)	6.02/(36, -48, 22)	38, -46, 25

**Table 4:** Group differences of FA and RD in the eigenvariate values within common clusters on smoothed data

DTI Measures	FA				RD (um^2^/msec)			
	
	Mean ± SD		%Diff	p	Mean ± SD		%Diff	p
						
	TD	DS			TD	DS		
Left FOF	0.309 ± 0.034	0.369 ± 0.038	19.5	< 0.001	0.683 ± 0.038	0.623 ± 0.027	-8.8	< 0.001
Right FOF	0.308 ± 0.022	0.377 ± 0.035	22.2	< 0.001	0.663 ± 0.024	0.602 ± 0.027	-9.4	< 0.001
Left FLS	0.329 ± 0.038	0.420 ± 0.048	27.6	< 0.001	0.679 ± 0.032	0.598 ± 0.045	-12	< 0.001
Right FLS	0.380 ± 0.026	0.444 ± 0.028	16.8	< 0.001	0.681 ± 0.037	0.602 ± 0.031	-11.5	< 0.001

**Table 5:** Group difference of MD and AD in the eigenvariate values within common clusters on smoothed data

DTI Measures	MD (um^2^/msec)				AD (um^2^/msec)			
	
	Mean ± SD		%Diff	p	Mean ± SD		%Diff	p
						
	TD	DS			TD	DS		
Left FOF	0.808 ± 0.030	0.768 ± 0.026	-4.9	0.001	1.058 ± 0.035	1.058 ± 0.037	0	0.494
Right FOF	0.788 ± 0.023	0.753 ± 0.020	-4.5	0.001	1.038 ± 0.035	1.056 ± 0.041	1.7	0.091
Left FLS	0.815 ± 0.030	0.763 ± 0.034	-6.4	0.001	1.088 ± 0.046	1.094 ± 0.058	0.65	0.349
Right FLS	0.847 ± 0.038	0.790 ± 0.026	-6.6	0.001	1.181 ± 0.054	1.168 ± 0.039	-1	0.237

### Correlations with Cognition

Our previous hypothesis of functionally significant neural connectivity differences between TD children and those with 22q11.2DS predicts such a relationship in the current findings. This is especially true because the common clusters are situated in bilateral parietal and frontal regions and we suggested that "a major cause of both the visuospatial and numerical cognitive deficits is a dysfunction in the posterior parietal lobes [p. 132 13] " of the 22q11.2DS group. Therefore, we correlated diffusivity values in the ROIs with performance data from a basic spatial attention task. In our "Cueing" task, a centrally presented spatial cue influences attentional orienting to one of two peripheral locations. Valid cues orient attention to the location of a subsequently appearing target while invalid cues orient attention the opposite location, requiring disengagement and re-orientation of attention before a response can be made [p. 132 13]. We calculated the "invalidity cost score" (ICS), which is the difference between response time on invalidly cued trials and validly cued trials. The larger the score, the greater was the impairment in spatial attention. Analyses of these data have been presented elsewhere [13], showing that children with 22q11.2DS exhibited much greater invalidity costs that are indicative of impaired spatial attention. Thus, if our hypothesis about the role of parietal dysfunction is correct we might well expect to find a relationship between the changes in connectivity that we detected and performance on this visuospatial attention task.

**Figure 5:**
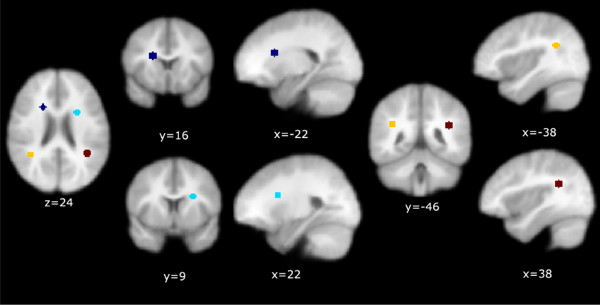
Location of spherical 5 mm fROIs used for correlation analyses.

Since the ROIs described in Table 2 and Figure 4 were quite extensive we created the equivalent of "functional ROIs (fROIs)" to avoid partially averaging across white matter tracts not relevant to this analysis. These fROIs were defined as spheres with an arbitrary radius of 5 mm centered on the Talairach coordinates of the peak z-scores of the most similarly located subclusters in the RD and FA maps (see Figure 5 and Table 2). We extracted the first eigenvariate values of each child's normalized DTI maps within these fROIs. Correlations with ICS using linear regression methods were performed. For this analysis alone we used unsmoothed DTI data to avoid alterations to the individual eigenvariates imposed by the use of a Gaussian kernel (or filter).

The results were striking, not least because they were again in opposing directions. Significant Pearson's r correlations showed that, for the 22q11.2DS group, FA was significantly positively correlated with ICS in the right parietal cluster (r = .78, p = .02) while for the TD group FA was significantly negatively correlated with ICS in the right frontal cluster (r = -.58, p = .03). This clearly indicates that higher parietal FA values were related to poorer performance on the task for the 22q11.2DS group. The more typical relationship, of higher FA to better performance, was found in the TD group, but only for the right frontal cluster. A more direct complement to RD is AD since the total MD is calculated from AD and RD combined. Not surprisingly, there were also significant correlations between ICS scores and AD values. For the 22q11.2DS group, AD was significantly positively correlated with ICS in both the left and right parietal clusters (r = .90, p < .001, r = .78, p = .02 respectively). In the TD group AD was also significantly positively correlated with ICS, but only in the left parietal cluster and to a weaker extent than in the 22q11.2DS group (r = .55, p = .004). This indicates that AD in these parietal clusters, to the extent that it exists, impairs spatial attentional function, presumably because it indicates connectivity to regions other than adjoining parietal cortex. No other correlations were statistically significant.

## Discussion

In this paper we have presented evidence indicative of opposing patterns of neural connectivity in the frontal and parietal lobes of children with 22q11.2DS and TD controls. These data take the form of commonly located clusters within major longitudinal fasciculi in which complementary measures that can be computed from the diffusion tensor produced patterns of differences that were essentially the opposite of one another in the two groups. Specifically, children with 22q11.2DS had significantly higher fractional anisotropy values in bilateral parietal and bilateral frontal clusters compared to TD control children. By contrast, TD children had significantly higher radial diffusion values in the same locations compared to children with 22q11.2DS. The high FA values in the 22q11.2DS group clusters suggest a pattern of connectivity that is primarily parallel to the major fiber tracts in which they are located. These are the superior longitudinal fasciculus for the parietal clusters and the frontal-occipital fasciculus for the frontal clusters (see Table 1 and Figure 3 for details). Conversely, the high values of RD in the TD control group clusters clearly indicate a pattern of connectivity that is greater in the perpendicular plane to the major fiber tracts in which they are located. We interpret this as evidence of greater connectivity with contiguous parietal and frontal cortical regions. It is also worth noting that in Figure 1b most of clusters with greater mean diffusivity in the TD than the 22q11.2DS group overlap their clusters of higher radial diffusion. Since mean diffusivity consists of both axial and radial diffusion, this finding is consistent with evidence of a more widespread pattern of greater branching from major white matter tracts into adjoining cortex in this group, or higher complexity in their connective patterns derived from the RD component of this measure. Based on our statistical analyses we made the straightforward inference that higher radial values probably indicated greater connectivity to contiguous parietal and frontal cortex in TD children than those with 22q11.2DS, and that the higher FA values in the 22q11.2DS group represented a reduction of this connectivity matrix.

We further reported significant, and similarly complementary, patterns of correlations of visuospatial attention scores to diffusivity values in focal sub-regions of those clusters. These results generate some important hypotheses about neural connectivity and their effect on cognitive function that we intend to explore in future studies. The apparently counter-intuitive relationship between higher factional anisotropy in the right parietal cluster and worse performance on the attention task in the DS22q11 group is explained to a degree by their correlations of axial diffusion and cognitive performance scores. Both parietal clusters showed extraordinarily high positive correlations indicating that higher axial diffusion was related to worse performance. At the same time, while higher FA had correlated with better performance for the TD group (albeit in the right frontal cluster), there was again a positive correlation of AD with invalid cue cost size in that group in the left parietal cluster. These results suggest that increasing amounts of axial diffusion in parietal clusters relate to poorer performance on visuospatial attention tasks. This is presumably because axial diffusion is the complement of radial diffusion and so may reflect reduced complexity in or degree of connectivity to critical surrounding cortex. The fact that children with 22q11.2DS had much higher FA values in their parietal (and frontal) clusters than typical controls was taken to indicate that their connectivity perpendicular to the major fasciculus into adjoining cortex was reduced. The correlations with AD, a purer measure of orientation along a single axis than FA, appear to confirm that impression. At the same time, even though radial diffusion, and thus inferred cortical connectivity, was significantly higher in TD controls for all clusters, there is still necessarily a component of total (mean) diffusivity that is axial in nature. Apparently, the more axial diffusion there is on these clusters the greater the negative impact it has on performance. Correlations were also carried out with performance data from higher-level numerical cognition tasks such as speeded enumeration of random dot patterns and analog and numerical magnitude comparison. While some results were significant and broadly consistent with those presented above, no strong pattern like the one just described was evident. Our interpretation is that, because tasks such as these depend on acquired knowledge and strategy choice to a much greater degree than is the case for simple spatial cueing of attention, the relationship between basic neurobiological measures and cognitive function is bound to be much weaker. However, Barnea-Goraly and colleagues [25] recently reported a similar correlation between FA and scores on a standardized Math test that suggest just such a relationship does exist in a slightly older group of participants with 22q11.2DS.

One question that is important to ask when interpreting neuroimaging findings in atypically developing populations is whether finding is more likely to indicate the delayed progression of typical development or that which arises from a completely different developmental trajectory [26]. It appears that the findings we report here are more likely to be an instance of the latter than the former. This is because a recent study [27] reported that the superior longitudinal fasciculus, where our most significant findings occurred, undergoes a prolonged maturation in typically developing individuals. In infancy and early childhood FA values are low and angle ± values are high, denoting reduced organization of fibers in the tract. That pattern reverses into the adult profile by 5 years of age. By contrast our developmentally delayed population of 7–14-year-old children with DS22q11 had higher FA values than our typically developing cohort in the cluster that we described, thus indicating that a completely different pattern of development had taken place. Whether this is likely to normalize over time can only be determined by future cross sectional or longitudinal studies involving wider age ranges. Interestingly, Hoeft et al. [28], reported very similar findings to ours in children with Williams syndrome. That is another disorder characterized in part by significant impairments in visuospatial ability and their findings of increased FA in the right superior longitudinal fasciiculus correlated with poorer scores on a standardized measure of visuospatial ability. Together, these findings do indeed appear to indicate that atypical connectivity in the longitudinal fasciculus is a biomarker of and associated with visuospatial cognitive impairments in at least two neurogenetic disorders.

Despite the apparent evidence in favor of our interpretation of the data presented, it must still be kept in mind that the precise neuroanatomical implications of diffusion tensor MRI data remain exceedingly difficult to evaluate. For example, Pierpaoli et al. [29] discuss in detail their conclusion that no clear relationship has been defined between measures of anisotropy or other diffusion measures and packing density of fibers, myelin density or distribution. In fact, Pierpaoli et al. conclude that they were "unable to identify a single microstructural factor or a combination of them to account for the observed differences in diffusion anisotropy in all regions of white matter in the normal human brain" (p. 646). However, some of the more direct measures of the directionality of the main axes of diffusion may have a sounder interpretive basis. For example, Song et al. [30] suggest that one interpretation of increased radial diffusion measures in some populations, such as those with multiple sclerosis, may actually be a signal of dismyelination. Only further analyses that include higher resolution data combined with cognitive performance data and other imaging measures will be able to converge upon a more definitive account of the patterns in and functional implications of neural connectivity in clinical populations such as the one described here. Our ongoing research program has just such goals and will resolve two of the major limitations of our current results by providing complete brain coverage and by acquiring data in 12 directions at higher resolution on a 3T scanner. These advances will allow us to examine connectivity patterns in our data in more detail, to generate reasonable visualization of differences that are detected, and also to relate them to a wider range of cognitive processes that are impaired in children with 22q11.2DS. In doing so we expect to further advance our understanding of the neural foundations of cognitive impairments in 22q11.2DS and to use that knowledge in the design of effective interventions.

## Conclusion

Several sources of data now point to alterations in neural connectivity as a key factor in understanding the visuospatial, and likely numerical, cognitive impairments exhibited by individuals with chromosome 22q11.2 deletion syndrome. Rather than a marker of developmental delay, these findings appear to indicate an atypical developmental trajectory for key neural networks that underlie such functions in typically developing individuals. As further research elucidates the exact nature of the structural and functional implications of such changes, it is likely that the results can be used to inform the design of targeted interventions that may reduce the severity of such impairments.

## Competing interests

The authors declare that they have no competing interests.

## Authors' contributions

TJS drafted the manuscript and supervised all management, analysis, and interpretation of the data. He also conceived of the study, secured funding, was responsible for its design and coordination, ZW, assisted by HZ and BA carried out the data analysis, produced all figures and helped to draft the manuscript, JCG supervised and conceived of the design of the advanced image processing methods, with BA and HZ. All three contributed to the manuscript. GTS was instrumental in developing the specific interpretation of the data in cognitive function terms and he contributed to the manuscript. All authors read and approved the final manuscript.
